# On the Diversity of Linguistic Data and the Integration of the Language Sciences

**DOI:** 10.3389/fpsyg.2017.02002

**Published:** 2017-11-23

**Authors:** Roberta D’Alessandro, Marc van Oostendorp

**Affiliations:** ^1^Utrecht Institute of Linguistics OTS, Utrecht University, Utrecht, Netherlands; ^2^Meertens Instituut (KNAW), Amsterdam, Netherlands; ^3^Centre for Language Studies, Radboud University Nijmegen, Nijmegen, Netherlands

**Keywords:** generative grammar, minimalism, biolinguistics, construction grammar, functionalism, cognition, linguistic data

## Abstract

An integrated science of language is usually advocated as a step forward for linguistic research. In this paper, we maintain that integration of this sort is premature, and cannot take place before we identify a common object of study. We advocate instead a science of language that is inherently multi-faceted, and takes into account the different viewpoints as well as the different definitions of the object of study. We also advocate the use of different data sources, which, if non-contradictory, can provide more solid evidence for linguistic analysis. Last, we argue that generative grammar is an important tile in the puzzle.

## Introduction

In a recent article, Christiansen and Chater (2017) (henceforth CC) argue in favor of an ‘integrated science of language.’ Just as “integration and interaction between levels of analysis and diverse data is ubiquitous [in] the physical and biological sciences,” progress in linguistics can only be guaranteed by taking into account a wide variety of data from a range of different sources.

We suspect there are not many linguists who would disagree with the observation that attempts to integrate knowledge and to facilitate interaction between students of language working at different ‘levels of analysis’ would probably be beneficial to the field. Clearly, the number and variety of empirical sources that have become available in recent decades for anyone interested in the topic of human language has broadened considerably, and continues to do so: from ultrasound measurements to automatic exploration of large amounts of words used on social media, and from fieldwork notes on Amazonian languages that are already extinct to neurolinguistics data on people learning artificial languages while in an MRI machine – all of these can potentially shed light on the question what human language is and how it works. It is regrettable indeed that the boundaries between the people studying all these different types of data are seldom crossed.

CC, however, see one major obstacle in this integration: ‘Chomskyan’ linguistics. They state: “Many of the phenomena that have become the focus of syntactic theory are so abstract that they are often difficult to connect even with specific linguistic phenomena, let alone with experiments on how people process language or observations of how children learn their native tongue.” For this reason, they propose replacing generative grammar with construction grammars (for which they cite Goldberg, 2006; strangely, they do not cite *any* reference for generative grammar), because their “quasi-regular nature […] allows them to capture both the rule-like patterns as well as the myriad of exceptions that often are excluded by fiat from the old view built on abstract rules.” They do not give precise details about how construction grammar makes better predictions than generative grammar.

The structure of CC’s argument is very similar to that put forward by Levinson and Evans (2010) (henceforth LE), although CC do not mention that earlier paper. LE state that “[generativists] draw on a very small subset of the data – especially, intuitions about complex clauses. Meanwhile, the available data types (corpora, typological databases, multimedia records), and the range of data over the languages of the world, has vastly increased in recent years, as has the scientific treatment of grammatical intuitions” and they contrast this with “the vastly increased quantity, quality and types of data now available to the descriptive and comparative linguist.” Like CC, LE seem to argue for an integrated science of language, in which everybody is welcome to contribute, except for the Chomskyans.

We believe that CC and LE misrepresent the range of methodologies that are used by scholars sympathetic to the generative paradigm, in which many kinds of data have also been studied recently, and sometimes with considerable success. We agree with them that the question of how the body of ideas that constitutes generative grammar should relate to the wealth of data that is available to us is important, as is whether there is any place for generative inquiry/biolinguistics (Jenkins, 2000) in an integrated science of language. We want to discuss both of these questions in this short contribution.

## The Ontology of Language in Generative Grammar

Anybody who seriously aims to undertake an integrated study of language should first note that there is very little agreement about the ontology of the object of study among linguists. One clear opposition is that which could be referred to as Chomsky vs. Saussure. In the first line of thought, language is seen as a cognitive object, something which resides in the mind of an individual speaker (Chomsky, 1957, 1965 *ff.*), and communities present chaotic mixtures of these idiolects. The other line is the Saussurean view (also foundational to, e.g., Labovian linguistics) in which language resides in a community, and the language production of individual speakers is an imperfect reflection of those speakers. Both of these positions seem coherent in their own right, and work from both schools can be combined, although they obviously conflict in their ultimate vision of what language is. There are also other visions available, such as the Platonic view (Postal, 2009) which sees language as “a purely abstract object, on a par with those of mathematics.”

It is important to point out that such approaches are not easily reconciled, as they seem incommensurable in the well-known sense of Kuhn (1962): they are different in scope. This does not mean that data or even insights cannot be transferred from one to the other; witness successful work that has been done over the years that shows otherwise (see for instance Kroch’s, 1994; Cornips and Corrigan’s, 2005; and Adger’s, 2016 work on “socio-syntax,” to use Adger’s term). Such interactions are, however, more complicated than different ‘levels of analysis’ (say, the subatomic level to the atomic level) in physics; the linguistic disciplines are simply not easily integrated in any reasonable sense of that word.

It is not clear where CC and LE stand in this debate about the ontology of language. On the one hand, there is a certain sympathy in both papers for so-called cognitive grammar (of which construction grammar is usually seen as a variant, i.e., *Cognitive Construction Grammar*, inspired by Goldberg, 1995 *ff.*), although both papers occasionally refer to ‘culture’ and ‘communication’ as sources of explanation, leaving open the question of how these different modalities relate to each other (whether they are to be seen as ‘different levels of analysis’). At first sight, the first victim of a revolutionary ‘integration’ along the lines of LE and CC seems to be the Saussurian/Labovian view of language rather than the Chomskyan view. In any case, there seems to be no attempt to reconcile these different views with one another, or with the Platonic view (but see Watumull, 2013 on the potential compatibility of Platonism and biolinguistics).

CC make use of a very salient metaphor: language is like a crossword, where figuring out one clue will help figure out the next clue. They describe the way that language acquisition takes place in a crossword-like fashion. Children are sensitive to “multiple sources of probabilistic information available in the linguistic input: from the sound of words to their co-occurrence patterns to information from semantic and pragmatic contexts.” According to CC, there is no need to postulate an innate set of pre-existing categories, for instance: children can infer categories from statistical analyses of distribution. The construction grammar approach accounts very well, CC maintain, for the diversity of the world’s languages.

The first observation that comes to mind is that this view of generative grammar is inaccurate: many generative approaches do not postulate pre-existing categories (see the work of Wiltschko or Biberauer on emergentist features). Then, it seems to us that construction grammar lacks predictive power: much like the old transformational grammar rules, in construction grammar everything goes, as long as there is evidence for it. No restriction is imposed on structures because of the system itself. We know that this is not accurate. Although many of the macro-parametric approaches have proved unsuccessful, some generalizations on co-occurring structural properties across languages cannot be easily denied.

Keeping the empirical coverage aside for the moment, we submit that, using CC’s metaphor, integration is impossible, because the clues are not for the same crossword. It is possible that convincing theories will be developed in which a link can be found between the psychological and the sociological, and between each of these and the abstract, in which case we could hope to build a truly integrative framework for the language sciences. None of this means that one particular view (of those mentioned) on this issue on this is inherently superior. As Chomsky (2001:34) phrased it:

Internalist biolinguistic inquiry [Chomsky’s term for what we call Chomskyan linguistics here] does not, of course, question the legitimacy of other approaches to language, any more than internalist inquiry into bee communication invalidates the study of how the relevant internal organization of bees enters into their social structure. The investigations do not conflict; they are mutually supportive. In the case of humans, though not other organisms, the issues are subject to controversy, often impassioned, and needless.

It should be added that Chomsky’s practice or that of his followers may not always have conformed to this dictum, and have sometimes suggested that the *only* way of doing linguistics is by doing generative grammar, or that ‘language’ is a synonym for ‘the innate capacity to acquire language.’

We propose, then, that rather than attempting a premature integration of different branches of linguistics, we should maximally profit from the *mosaical* nature of the field: the many different viewpoints that are taken on subject matters that have many things in common. Integration, as proposed in CC and LE, would lead to severe impoverishment of those points of view, forcing all linguistics to work in one frame (construction grammar) that was never designed to answer all questions and that has not had the time to be sufficiently tested. To borrow another set of terms from Kuhn, it is as if CC and LE want to move immediately from a period of (perceived) crisis to normal science, without wanting to go through the stage of paradigm shift. We think linguistics is not yet ready to be a coherent normal science, and it would be detrimental to pretend that it is: one can obviously always carry out numerous ‘empirical studies’, but without a solid base it is impossible to achieve the kind of cumulative effect that is so typical of ‘real science.’

Generative grammar, or more precisely a form of biolinguistics, based on a view in which language is primarily an internal tool for thought or expression of thought, cannot be excluded from such a multifaceted way of studying language. One can argue, if one sees reasons to do so, that current work on this matter is not satisfactory or is even wrong, but one cannot *a priori* deny that there are reasons to engage in such an enterprise.

A mosaical view on linguistics, we find, is a better metaphor than a crossword: we have tiles of different shapes, different colors, and differing importance. Inserting one tile in the mosaic will only give us a clue about what comes next, what is adjacent. Only the combination of all tiles allows us to see the full picture. If some tiles are missing, we will be able to figure them out. But, importantly, tiles do not resemble crossword clues, as they are not uniform in nature. Insights from different disciplines can all contribute tiles. The combination of all these tiles, including those regarding structural dependencies coming from generative grammar, will give us a picture of language.

## The Data for Generative Grammar

This, then, seems to us the most reasonable position for generative grammar among the language sciences: as an approach to understanding what is specific about human language (in particular syntax) and to specifying what computational capacity the human mind needs to be able to acquire and use syntax. In no way should this prevent generative grammarians from collaborating with scholars working on other aspects, sometimes even within a completely different paradigm. We have already mentioned above work on the crossroads with sociolinguistics above, but we should also consider work such as that by Andrea Moro on neurolinguistics, by George Walkden and David Lightfoot on diachronic linguistics, and by William Snyder, Maria Teresa Guasti, and Jason Rothman on psycholinguistics and acquisition.

It follows from this list that CC and LE’s view of the range of types of data on which generative work is based is too pessimistic. There is also no reason why it could not widen more. For instance, the fact that intuitions often lack a quantitative component does not make them inherently less valuable, as Labov (1987), one of the fathers of quantitative linguistics, reminds us:

But the qualitative is not easily displaced. Many forms of linguistic behavior are categorically invariant. Furthermore, the number, variety and complexity of linguistic relations are very great, and it is not likely that a large proportion can be investigated by quantitative means. At present, we do not know the correct balance between the two modes of analysis.

On the contrary, any kind of scientific enterprise can only benefit from including as much empirical evidence as possible. As the eventual goal of generative grammar is to discover properties of the human mind, there is no such thing as direct evidence for this; there is no golden path. Intuitions have the advantage of being cheap and easy to acquire, but since they have their own inherent problems (they are not always as clear as we would want them to be; there can easily be interference with external norms on language, etc.), it seems that extending the empirical basis can only be a good thing.

For this we could follow, for instance, the taxonomy offered in van Oostendorp (2013), which was made for phonology, but can be easily extended to syntax: this taxonomy recognizes four types of evidence: traditional evidence (such as judgments, or the Wug tests); experimental evidence (such as that acquired in psycholinguistic of neurolinguistics laboratories); evidence from large databases and corpora (whether found in historical archives or tagged collections of modern text); and formal evidence (the results of computer modeling, analysis of formal elegance, etc.). All of these general types of data can be helpful beyond what we can establish from judgments alone. For instance, artificial language learning experiments (Moro, 2016) have shown that ‘crazy patterns,’ predicted not to exist by current theories, involve a different part of the brain than ‘realistic patterns.’ Automatic searching of large corpora can lead us to find patterns that an analyst would never have thought of independently. Computer modeling helps to make theories maximally explicit and thereby exposes hidden flaws.

None of these data can give us direct access to what we are really interested in – an object of considerable abstractness. We can therefore only aim to find convergent evidence from many different sides. The work on these types of data can of course take place in cooperation with researchers with a slightly different focus, which can in fact improve the way we approach the object of study. It does not necessarily mean that one has to share the same view on what should be studied.

Finally, it should also be kept in mind that even people who consider themselves practitioners of Chomskyan generative syntax do not necessarily have the same interests. We feel that there is a rather wide consensus that there are at least two types: those working in some version of what used to be called Government and Binding Theory (Chomsky, 1981), taking an interest mostly in trying to explain patterns in individual language varieties; and those subscribing whole-heartedly to the Minimalist Program (Chomsky, 1995). The former will typically be closer to types of data such as those just listed, whereas for the latter, the analyses formed by G&B count as data of some kind. This is the kind of work that presumably led CC to their complaint that the analyses are “so abstract that they are often difficult to connect even with specific linguistic phenomena.” We hope to have shown by now that this vision is too narrow, as it presupposes that there is some non-theoretical way of deciding what “specific linguistic phenomena” are. However, all ‘linguistic phenomena’ are theory-laden and dependent on one’s ontology of language. Suggesting otherwise, and operating on the assumption that we have some pre-theoretical conception of the subject matter is, in our view, not going to lead linguistics very far.

## Conclusion

As sympathetic as it may sound at first sight, calls for ‘integration’ of the language sciences, such as those by CC and LE, do not take into account the fact that there is no consensus on what linguistics is about, or what the explananda are – and therefore what the data to be taken into account are. Rather than calling for an integration of this type, which in our view can only lead to multiple small case studies, and experiments without sufficient loopback to a strong theory, we think it is better to opt for a model of the language sciences as a mosaic of different views and methodologies, hoping that in this way – and by cooperating across the disciplines rather than dismissing some of them out of hand – we can achieve a better understanding of the multifaceted phenomenon that is human language.

## Author Contributions

All authors listed have made a substantial, direct and intellectual contribution to the work, and approved it for publication.

## Conflict of Interest Statement

The authors declare that the research was conducted in the absence of any commercial or financial relationships that could be construed as a potential conflict of interest. The handling Editor declared a past collaboration with the author RD.
